# Supplementary material for: Pyridine Derivatives—A New Class of Compounds That Are Toxic to E. coli K12, R2–R4 Strains
Source: Materials (Basel). 2021 Sep 18;14(18):5401. doi: 10.3390/ma14185401 (PMC8467192; doi:10.3390/ma14185401)
Supplement: Supplementary file 1 [file materials-14-05401-s001.zip › materials-1323899-supplementary.pdf]

Pyridine Derivatives—A New Class of Compounds that are Toxic to *E. coli* K12, R2–R4 Strains

Dominik Koszelewski [1], Ryszard Ostaszewski [1], Paweł Śmigielski [1], Anastasiia Hrunyk [1], Karol Kramkowski [2], Łukasz Laskowski [3], Magdalena Laskowska [3], Rafał Lizut [4], Mateusz Szymczak [5], Jacek Michalski [6], Kamil Gawin [6] and Paweł Kowalczyk [6,*]

[1] Institute of Organic Chemistry PAS, Kasprzaka 44/52, 01-224 Warsaw, Poland; d.koszelewski@icho.edu.pl (D.K.); r.ostaszewski@icho.edu.pl (R.O.); p.smigielski@icho.edu.pl (P.Ś.); a.hrunyk@icho.edu.pl (A.H.)
[2] Department of Physical Chemistry, Medical University of Bialystok, Kilińskiego 1 Str., 15-089 Białystok, Poland; kkramk@wp.pl
[3] Institute of Nuclear Physics Polish Academy of Sciences, PL-31342 Krakow, Poland; lukasz.laskowski@ifj.edu.pl (Ł.L.); magdalena.laskowska@ifj.edu.pl (M.L.)
[4] The John Paul II Catholic University of Lublin, Institute of Mathematics, Informatics and Landscape Architecture ul. Konstantynów 1 H, 20-708 Lublin, Poland; lizut@kul.pl
[5] Department of Molecular Virology, Institute of Microbiology, Faculty of Biology, University of Warsaw, Miecznikowa 1, 02-096 Warsaw, Poland; mszymczak@biol.uw.edu.pl
[6] Department of Animal Nutrition, The Kielanowski Institute of Animal Physiology and Nutrition, Polish Academy of Sciences, 05-110 Jabłonna, Poland; j.michalski@ifzz.pl (J.M.); k.gawin@ifzz.pl (K.G.)
* Correspondence: p.kowalczyk@ifzz.pl

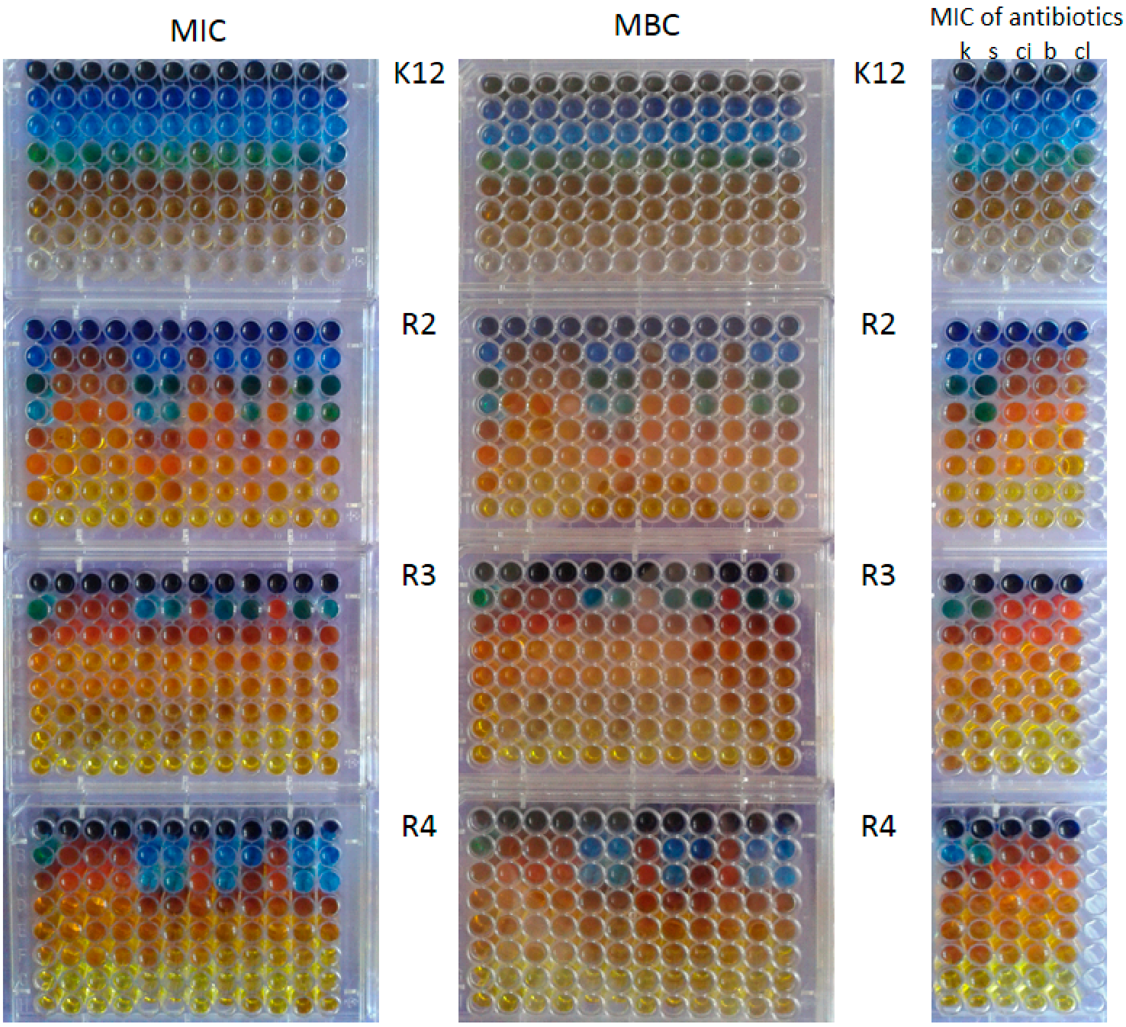

Figure S1. Examples of MIC and MBC on microplates with different concentration of studied compounds (μg/mL^{-1}). Resazurin was added as an indicator of microbial growth with K12, R2, R3 and R4 strains with tested 11 compounds. as described in Table 2. Additionally examples of MIC with different strains K12, R2, R3, R4 of studied antibiotics with kanamycin (k), streptomycin (s), ciprofloxacin (ci), bleomycin (b), cloxacillin (cl) in (μg/mL^{-1}).

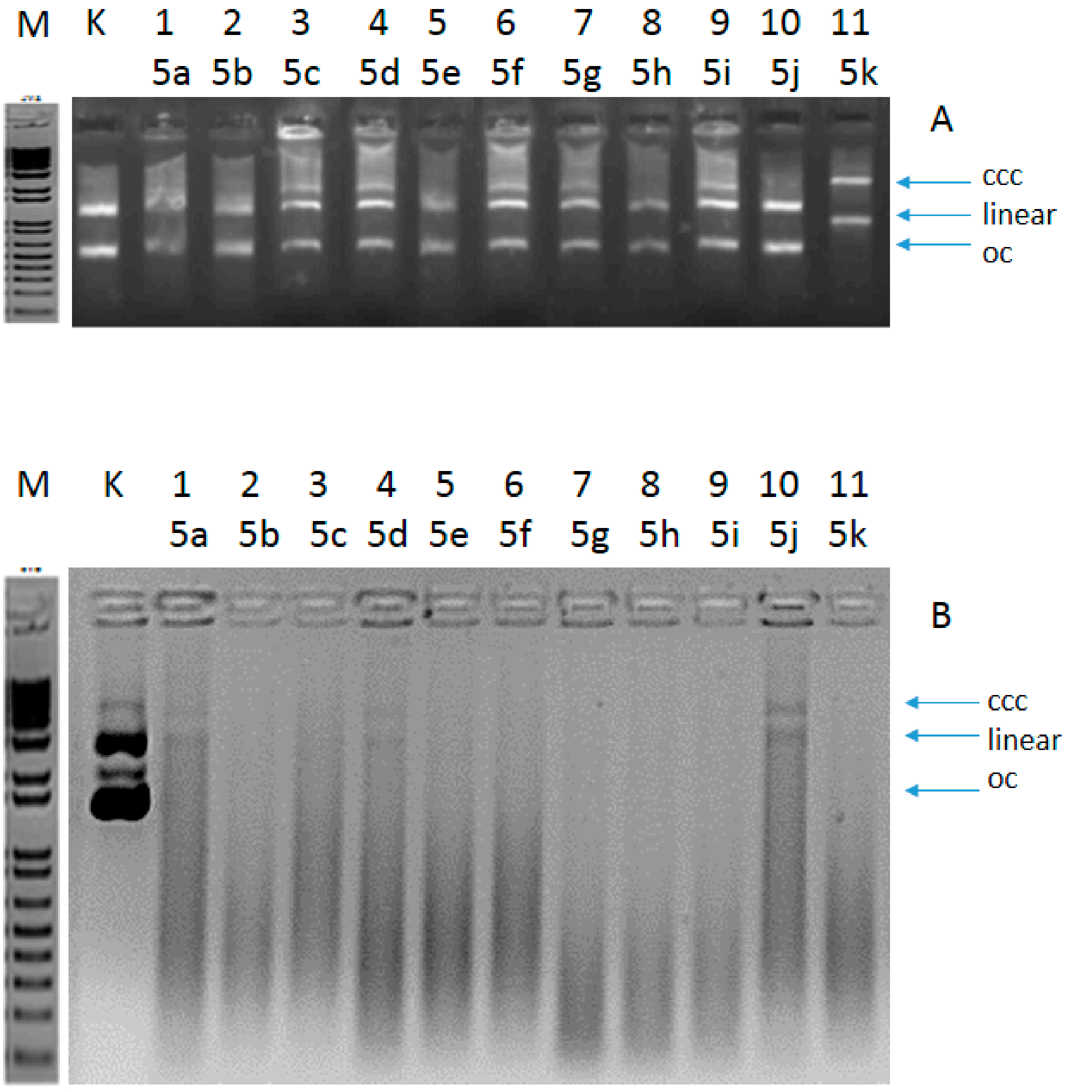

Figure S2. An example of an agarose gel electrophoresis separation of isolated plasmids DNA on R4 strains modified with selected pirydine derivatives (Panel A) from 1-11 as described in Table 2, digested with repair Fpg protein (Panel B). M-marker.

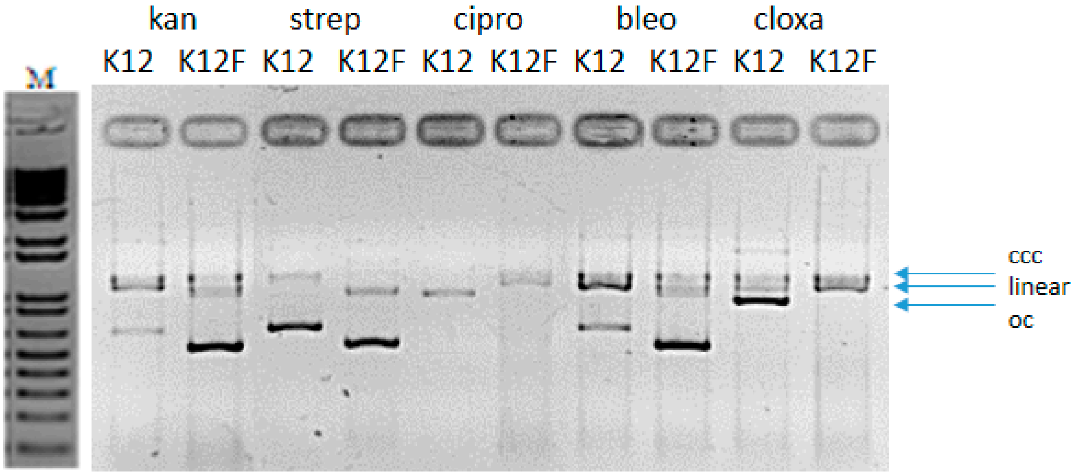

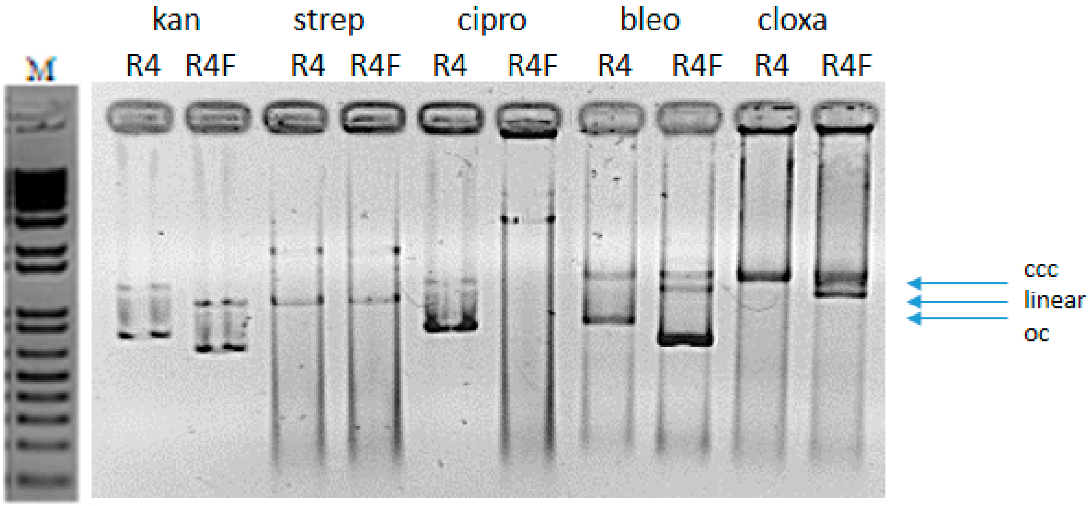

Figure S3. An example of an agarose gel electrophoresis separation of isolated plasmids DNA from K12 and R4 strains modified with antibiotics: kanamycin, streptomycin, ciprofloxacin, bleomycin and cloxacillin digested (or not) with repair enzymes Fpg. M-marker.
